# Perceptions of the Role of Diet Among People With Constipation: Dietary Contributors and Relievers to Symptoms and Research Priorities

**DOI:** 10.1111/jhn.70201

**Published:** 2026-01-21

**Authors:** Dominic N. Farsi, Kevin Whelan, Eirini Dimidi

**Affiliations:** ^1^ Department of Nutritional Sciences King's College London London UK

**Keywords:** attitudes, constipation, diet, foods, perceptions, research priorities

## Abstract

**Background and Aims:**

Diet is a potential management option for constipation; however, people's perceptions regarding its role and their dietary behaviours are unclear. The aim of this study was to explore the perceptions of the role of diet in constipation, including dietary contributors and relievers of constipation symptoms, and the attitudes towards future dietary research in constipation, in people with self‐reported constipation.

**Methods:**

An online questionnaire was completed by adults with self‐reported constipation based in the United Kingdom. Participants responded to questions on constipation symptoms, perceptions of the role of diet in constipation, perceived dietary contributors and relievers, and future research priorities. For perceived contributors and relievers, the survey included a list of 143 foods/beverages to rate the perceived impact on constipation symptoms using a Likert scale.

**Results:**

Overall, 204 individuals (mean age 44 (SD 16.2) y, 91.7% female) completed the questionnaire. In total, 94% (184/204) of respondents believed diet plays an important role in constipation, and 88% (180/204) would prefer effective dietary options over medication for managing symptoms. The most frequently reported dietary relievers of constipation symptoms were prunes (85%), dates (71%), prune juice (70%) and beans (70%), while white bread (69%), refined sugary breakfast cereals (61%), processed red meat (60%) and cake (57%) were the most frequently reported contributors to constipation symptoms.

**Conclusions:**

The survey highlights that most people with constipation believe diet is important for the management of constipation. Given the wide range of relievers and contributors, research on interventions that include whole dietary patterns is warranted.

## Summary

- Most people with constipation believe diet plays an important role in constipation and prefer adopting dietary management approaches over using medication.
- Most people with constipation believe more research is needed in diet and constipation, with a particular focus on investigating whole dietary approaches.
- Prunes, dates and prune juice are perceived to be the top constipation relievers, while white bread, refined sugary breakfast cereals and red processed meat were the top constipation contributors.

## 1 Introduction

Constipation is a problematic, often chronic condition, carrying a substantial burden for individuals through bothersome symptoms that have a negative impact on quality of life [1, 2]. It is estimated that chronic constipation affects 11.7% of the global population [3], although prevalence can vary from 10% to 15%, depending on the country and the Rome criteria applied [1]. As well as at an individual level, constipation also elicits economic burden to healthcare systems [4, 5]. Considering the high levels of treatment dissatisfaction associated with constipation [6, 7], more effort is required to design research to delineate efficacious treatments. Amongst treatments, diet is well acknowledged to play a substantial role in the management of constipation [8]. However, several challenges and unknowns persist in the dietary management of constipation.

First, evidence from clinical trials on the effect of foods, drinks and diets in treating chronic constipation is scarce [9]. Clinical guidelines for constipation commonly offer dietary recommendations as a first‐line management option, but these are limited in number, may make recommendations not supported by a strong evidence base, and may not reflect the dietary behaviours or preferences of people with constipation [10, 11, 12]. More recently, international guidelines specifically for the dietary management of constipation have been published, highlighting the growing importance of diet in the constipation management algorithm [13, 14]. Second, the limited existing clinical trials of diet tend to focus primarily on constipation treatment, rather than prevention or long‐term maintenance, which are areas that may be important to patients and to public health. Third, it is important that interventions and outcomes explored in clinical research align with what patients themselves consider meaningful.

Understanding people's perceptions and attitudes on the dietary management of constipation can provide valuable insights for future research of new management options, because they can inform underexplored but commonly used dietary strategies. For example, kiwifruit had been traditionally used in some countries to relieve constipation for over half a century, despite a lack of scientific evidence, and yet it wasn't until the 2000s that the first clinical trial was conducted [15], followed by multiple RCTs demonstrating the effectiveness of kiwifruit in improving constipation [9]. This case highlights the potential of patient‐reported perceptions to guide research toward effective but previously unexplored interventions. This patient‐informed approach can support the design of future trials that are more aligned with patient needs and experiences, ultimately leading to more effective and acceptable management strategies.

The aim of the present study was to explore (i) the perceptions of the role of diet in constipation, including dietary contributors and relievers of constipation symptoms, and (ii) attitudes towards future dietary research in constipation, in people with self‐reported constipation.

## 2 Materials and Methods

This was a cross‐sectional survey using an online self‐administered questionnaire. Ethical approval was granted by King's College London Research Ethics Committee on 18/06/2024 (LRS/DP‐23/24‐42020).

### 2.1 Participants

Members of the general population with constipation were recruited to participate in the survey. The inclusion criteria were adults ≥18 years of age who self‐reported as having constipation and lived in the United Kingdom. Exclusion criteria included self‐reported medical diagnosis of any of the following: irritable bowel syndrome, chronic diarrhoea (e.g., irritable bowel syndrome with diarrhoea), coeliac disease, Crohn's disease, ulcerative colitis, gastrointestinal cancer (e.g., stomach, bowel); history of major bowel surgery (except appendectomy or cholecystectomy); and currently pregnant or breastfeeding. Screening of participants against these criteria was integrated into the beginning of the online questionnaire survey, following consent, with only eligible consenting participants continuing to the main sections of the survey. Participants were recruited via circular emails to UK institutions and volunteer databases.

### 2.2 Questionnaire

A questionnaire was developed to investigate the perceptions of the role of diet in constipation. The self‐administered questionnaire was completed via Qualtrics (www.qualtrics.com) and included closed‐ended questions and free‐text boxes, comprising of four sections: (i) constipation‐related questionnaire; (ii) importance of diet in constipation and research targets; (iii) perceptions of contributors and relievers of constipation symptoms, and (iv) demographic characteristics (sex, ethnicity, education, and United Kingdom location).

### 2.3 Constipation‐Related Questionnaire

Participants responded to questions related to constipation, including the presence/absence of self‐reported constipation, duration of constipation, and whether they had received a formal diagnosis of constipation by a medical doctor. The presence/absence of chronic functional constipation was determined using the Rome IV criteria, although this did not serve as an inclusion criterion [7]. Information for each criterion was assessed using the Bristol Stool Form Scale for usual stool frequency and consistency [16], and questions regarding specific symptomology (e.g., “needing to strain to pass a stool for more than a quarter of your bowel movements”).

### 2.4 Importance of Diet in Constipation and Research Targets

Participants responded to questions concerning preferred approaches they use to manage their symptoms (e.g., supplements, individual foods, whole diet approaches, over the counter and prescription laxatives, biofeedback therapy, psychological support), whether they perceived diet plays an important role in constipation, and the potential role diet can play in constipation (e.g., cause, prevention, treatment, maintenance). The survey also asked about their perceptions of research on diet in constipation, including the amount of research conducted, and the focus of this research, including outcome measures used and interventions tested.

### 2.5 Perceptions of Dietary Contributors and Relievers of Constipation Symptoms

Participants were provided a curated list of 143 foods and beverages categorised into food groups to aid responses (e.g., fruit, vegetables, nuts and seeds, starchy foods and cereals, meats, etc.). Each food was rated using a 5‐point Likert scale for its perceived impact on constipation symptoms for that participant (‘significantly relieves’, ‘slightly relieves’, ‘no effect’, ‘slightly worsens’, ‘significantly worsens’) together with an ‘I do not know’ response for cases where the participant had not consumed or did not know the effect of the food on constipation. Responses from the 5‐point Likert scale were collapsed into three categories for ‘relievers’ (significantly or slightly relieves), neutral (no effect) and ‘contributors’ (significantly or slightly contributes) for each food and beverage.

### 2.6 Demographic Characteristics

Data were collected on demographic characteristics, including sex and ethnicity (using UK government standardised questions), gender, ethnicity, age, educational attainment, and location in the UK.

### 2.7 Statistical Analysis

Normality of the data was tested via Shapiro–Wilk tests and visual inspection of Q‐Q plots and histograms. Data were reported using descriptive statistics, with counts and percentage of study population calculated and reported for all questions. Means and standard deviations (SD) were reported for normally distributed data, and median and interquartile ranges (IQR) for non‐normally distributed data, when applicable. To explore how respondents varied in the number of foods they associated with constipation symptoms, responses from the 5‐point Likert scale were collapsed into two categories for ‘relievers’ and ‘contributors’ for each food and beverage. For each participant, the total number of foods selected in each group was calculated. Means and SD were computed to summarise these counts across all participants. Linear regression models were developed to examine whether demographic factors, including age, gender, constipation duration, ethnicity, education, medical diagnosis of constipation and Rome IV classification, could predict the number of foods rated as contributing to, or relieving constipation symptoms. A *p*‐value < 0.05 defined statistical significance in the models. Statistical analysis was performed using R version 4.3.3 (R Foundation for Statistical Computing, Vienna, Austria).

## 3 Results

### 3.1 Study Population

In total, 204 people with self‐reported constipation completed the full survey (mean age 44 (SD 16.2)) (Supplementary Figure 1). Of the study population, 95% (193/204) resided in England, 91.7% were female (187/204), 66% (136/204) of white ethnicity, while the most reported highest qualification attained was a university degree level with 41.7% (85/204) of respondents (Table 1).

**TABLE 1:** Characteristics of 204 respondents with self‐reported constipation.

Variable	
Age, *mean [SD]*	44 [16.2]
Min ‐ Max	18–83
Gender, *n* (%)	
Female	187 (91.7%)
Male	17 (8.3%)
Number of years with constipation, *n* (%)	
1–5	76 (37%)
6–10	50 (25%)
11–15	21 (10%)
16–20	4 (2%)
20+	53 (26%)
Constipation status, *n* (%)	
Self‐reported	204 (100%)
Diagnosis by a medical doctor	61 (29.9%)
Meeting Rome IV criteria for functional constipation	118 (57.8%)
Ethnicity, *n* (%)	
Asian or Asian British	47 (23%)
Black, Black British, Black Welsh, Caribbean or African	12 (6%)
Mixed or Multiple ethnic groups	7 (4%)
White	136 (66%)
Other ethnic group	2 (1%)
Highest educational attainment, *n* (%)	
No formal qualifications	1 (0.5%)
Vocational qualifications	13 (6.4%)
School‐level qualifications	52 (25.5%)
University degree (e.g., BSc, BA)	85 (41.7%)
Postgraduate degree (e.g., MSc, PhD)	53 (26.0%)
Location in the United Kingdom, *n* (%)	
England	193 (95.0%)
Wales	3 (1%)
Scotland	8 (4%)
Northern Ireland	0 (0.0%)

### 3.2 Characteristics of Constipation

Although all participants self‐reported having constipation, only 58% (118/204) fulfilled the Rome IV diagnostic criteria for chronic constipation, and 30% (61/204) reported having a formal medical diagnosis of constipation. Most respondents reported having constipation for 1–5 years (76/204, 37%), while over a quarter reported having constipation for over 20 years (53/204, 26%) (Table 1).

**FIGURE 1:**
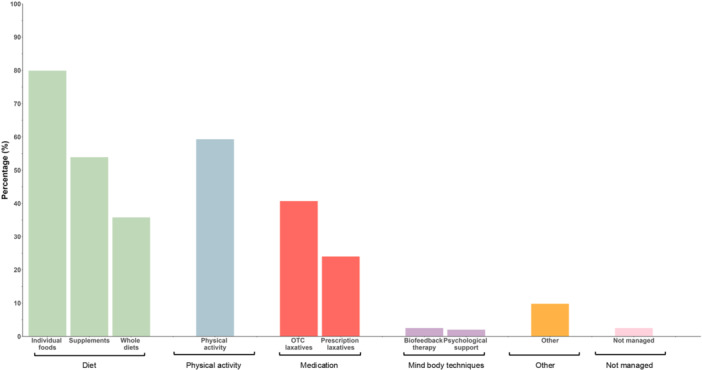
Approaches used to manage constipation symptoms. Approaches grouped into wider themes: diet, physical activity, medication, mind‐body techniques, other, and not managed.

On average, respondents reported three (SD 1.3, range 1–7) approaches in managing their constipation symptoms, with all responders reporting at least one preferred approach. Individual foods were the most commonly reported preferred approach to manage symptoms (80%), followed by physical activity (59%), dietary supplements (54%), and over the counter laxatives (41%). Most respondents (184/204, 91%) reported using at least one dietary strategy (i.e., supplements, individual foods, whole diet) to manage symptoms. The preferred approaches to manage constipation symptoms among survey respondents are presented in Figure 1.

### 3.3 Attitudes Towards Constipation and the Role of Diet

Notably, 94% (191/204) of respondents believed that diet played an important role in constipation. Most participants believed that diet can cause (158/204, 78%), prevent (150/204, 74%), maintain (150/204, 74%), treat (142/204, 70%) and worsen (132/204, 65%) constipation symptoms (Table 2).

**TABLE 2:** Perceptions and attitudes of people with self‐reported constipation towards the role of diet in constipation (*n* = 204).

	*n* (%)(*n* = 204)
Diet plays an important role in constipation	191 (94%)
Diet can play a role in:	
Causing constipation	158 (78%)
Preventing constipation from occurring	150 (74%)
Treating symptoms in those who have constipation	142 (70%)
Maintaining relief for those who have treated constipation symptoms	150 (74%)
Worsening constipation symptoms	132 (65%)
No role in constipation	7 (3%)
In a scenario where both effective dietary and medication approaches were available:	
Prefer diet over medication	180 (88%)
Prefer medication over diet	24 (12%)
Reason for preferring dietary approaches over medication:	
Diet is a “natural” way to manage constipation	164 (80%)
Diet can have benefits for overall health and not just constipation	165 (81%)
Diet avoids the side effects that constipation medication may have	132 (65%)
Diet does not have the negative long‐term health impact that constipation medication may have	118 (58%)
Diet is more economical than medication	80 (39%)
Diet enables people to self‐manage constipation symptoms better	116 (57%)
Other	7 (14%)

In a scenario where efficacious treatment options were available for both diet and medications, 88% (180/204) of respondents indicated that they would prefer diet over medications, with the benefits for ‘overall health’ (81%) and diet being a ‘natural way to manage symptoms’ (80%) selected as the most common reasons for preferring dietary approaches over medications (Supplementary Figure 2).

### 3.4 Perspectives on Future Research in Diet and Constipation

Overall, 90% (184/204) believed that more research is required in diet and constipation, particularly on the role of diet in treating (145/204, 71%), preventing (138/204, 67%), causing (129/204, 63%) and worsening (100/204, 49%) constipation symptoms (Table 3).

**TABLE 3:** Perspectives of people with self‐reported constipation regarding future research directions on diet in constipation (*n* = 204).

	*n* (%)(*n* = 204)
More research is required on diet and constipation	184 (90%)
Aspect of constipation that research should address through diet	
Causing constipation	129 (63%)
Preventing constipation from occurring	138 (68%)
Treating symptoms in those who have constipation	145 (71%)
Maintaining relief of symptoms for those who have treated constipation symptoms	130 (64%)
Worsening constipation symptoms	100 (49%)
Believe no role of diet in constipation	2 (1%)
Dietary intervention to be investigated in future research:	
Supplements (e.g., vitamins, minerals, probiotics, prebiotics)	116 (57%)
Individual foods (e.g., prunes, cereals, etc.)	142 (70%)
Whole diet patterns (e.g., Mediterranean diet, high fibre diet)	143 (70%)
Other	6 (3%)

**FIGURE 2:**
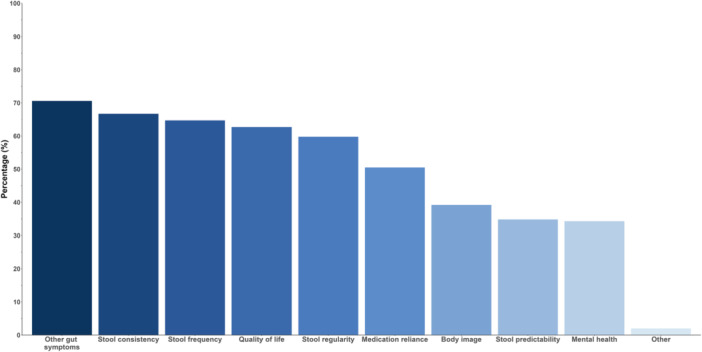
Respondent preference for outcomes to be investigated in future research studies in diet and constipation.

In terms of the outcomes of interest for future research, ‘other symptoms’ (e.g., straining) was the most commonly reported desired outcome (144/204, 71%), followed by stool consistency (136/204, 67%), stool frequency (132/204, 65%), stool regularity (122/204, 60%), and quality of life (128/204, 63%) (Figure 2).

Most participants were in favour of whole dietary patterns (70%) and individual foods (70%) over supplements (57%), to be investigated in future research (Table 3).

### 3.5 Dietary Relievers and Dietary Contributors to Constipation Symptoms

Of the 143 food and beverage items presented, on average, people reported 18.7 (17.3) to be contributors to constipation and, on average, 50.7 (31.4) to be relievers of constipation.

Linear regression models exploring the influence of demographic factors on the number of dietary relievers and contributors for constipation revealed that constipation duration, gender, ethnicity, educational attainment, medical diagnosis of constipation and Rome IV classification were not associated with the number of dietary contributors nor relievers to constipation (Supplementary Table 1). Only age was associated with the number of dietary contributors (*p* = 0.002) (Supplementary Table 1), with respondents aged 18–24 years reporting more dietary contributors (mean 26, SD 18.4) compared to respondents aged 55–64 years (mean 11, SD 9.6, *p* = 0.001) and compared to respondents aged 65+ years (mean 13, SD 10.5, *p* = 0.015) (Supplementary Table 2).

**FIGURE 3:**
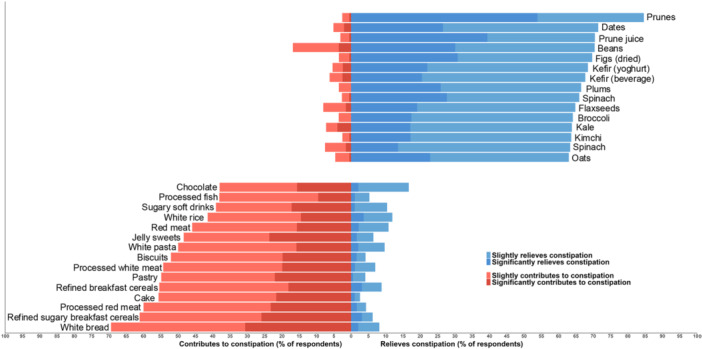
Top 15 most commonly perceived dietary relievers and contributors. Blue indicates reliever and red contributor, with darker shading representing the percentage of responses for having a ‘significant’ effect and lighter shades a ‘slight’ effect.

The most frequently reported dietary relievers of constipation symptoms were prunes (85%), dates (71%), prune juice (70%), beans (70%), figs (70%), kefir yoghurt (68%) and kefir beverage (68%), while the most frequently reported contributors to constipation symptoms were white bread (69%), refined sugary breakfast cereals (61%), processed red meat (60%), cake (56%) refined breakfast cereals (55%) and pastry (55%). The top 15 most commonly reported dietary relievers and contributors to constipation symptoms are presented in Figure 3, and the full list of the 143 foods and beverages included in the survey, along with the percentage of responses for the six different categories, is included in Supplementary Table 3.

## 4 Discussion

This study aimed to explore (i) the perceptions of the role of diet in constipation, including dietary contributors and relievers of constipation symptoms, and (ii) attitudes towards future dietary research in constipation, in people with self‐reported constipation. It confirms that almost all people with constipation believe diet plays an important role in their constipation, and highlights the foods and drinks they perceive to relieve or contribute to their constipation symptoms. The study also outlines dietary research priorities in constipation, with an identified need for more studies on whole dietary patterns and individual foods, and a preference on assessing gut symptoms such as straining as an outcome of interest. These findings are novel, as to our knowledge, perceptions concerning these topics have not been previously reported for people with constipation.

Overall, 94% of people with constipation believed that diet plays an important role in constipation, which is also evident by the fact that 91% have used at least one type of dietary approach to manage their condition. In fact, 88% of respondents preferred using dietary approaches over medication to manage their symptoms. The most cited reasons were that dietary strategies could improve constipation as well as overall health, and also that diet was a more ‘natural’ way of managing symptoms than medication. The perception that diet is a ‘natural’ management strategy unlikely to cause harm has been previously reported in qualitative studies of people with constipation symptoms [17, 18]. These perceptions highlight the value patients place on dietary interventions and suggest that these align more closely with their personal health and wellbeing goals. Therefore, effective dietary approaches ought to be favoured where possible over medication, and should be prioritised in both clinical care and future research.

Most people (80%) used individual foods (e.g., prunes) to manage their symptoms, while only about a third had adopted whole dietary approaches (e.g., high fibre diet). This behaviour mirrors the current evidence base with a systematic review identifying 17 RCTs investigating individual foods and drinks in constipation management, but only one trial evaluating a whole dietary approach [9]. Neither the behaviour of people with constipation nor the evidence base, both of which support individual foods over whole dietary patterns, align with most of the medical guidelines, which typically recommend broader dietary pattern modifications, particularly increased dietary intake of fibre as a first‐line management strategy [19]. This preference for individual foods may reflect their greater practicality and ease of implementation for people with chronic constipation and aligns with recent British Dietetic Association dietary guidelines, which recommend specific foods (e.g., kiwifruits, prunes, rye bread) for the management of constipation but do not provide recommendations for whole dietary approaches due to insufficient evidence [13, 14].

This discordance between patient behaviours, clinical evidence and medical guidelines highlights a critical gap in the evidence for whole diet approaches. Importantly, participants themselves recognised this need, with the majority expressing a preference for future research to focus on whole dietary patterns and individual foods, while fewer than half supported investigating supplements. Previous systematic reviews have identified 58 RCTs on fibre, prebiotic, probiotic, synbiotic, mineral, and food supplements, but only 17 RCTs on foods, drinks, and whole diets [9, 20, 21, 22], underscoring the imbalance in the research previously conducted. There is a clear need to realign research efforts with both clinical recommendations and patient interests, particularly by increasing the evidence base for whole dietary strategies in constipation management, for which there was only one RCT identified in a recent systematic review [9].

Physical activity was the second most reported approach for symptom management by respondents. Evidence suggests that physical activity may relieve constipation symptoms [23], and represents another approach which can also carry holistic benefits to health and well‐being beyond constipation management [24]. This highlights the potential value of investigating multi‐component lifestyle interventions that combine both dietary changes and physical activity for more effective constipation management.

Prunes, dates, prune juice, beans and figs were the most frequently reported constipation relievers. Prunes have been shown to be as effective as psyllium supplements in improving stool consistency and straining in people with chronic constipation [9]. While no studies have investigated the effect of dried figs in constipation, a RCT assessed the impact of fig paste compared to placebo and resulted in significantly softer stool consistency [9]. However, no RCTs exist on dates or beans in chronic constipation and, thus, their effectiveness is unknown. However, a cross‐sectional study of 13,945 participants of the US National Health and Nutrition Examination Survey showed that higher intake of total fruits and beans led to lower odds of constipation [25]. Kiwifruits, which have been investigated in multiple RCTs demonstrating they are as effective as psyllium supplements in improving multiple constipation outcomes [9], were reported as constipation relievers by only 56% of respondents. These findings highlight that commonly perceived dietary relievers are not always supported by a strong evidence base, and conversely, that some evidence‐based options are not widely recognised or used by those with constipation. This underscores the need for both further clinical research on under‐investigated foods and more effective knowledge translation strategies to align public understanding with current scientific evidence. It has previously been shown that, indeed, a factor in individuals trying certain remedies is driven by an awareness that such approaches have been tested in research, such as probiotics [26].

Our survey identified fermented foods such as kefir (both yoghurt and beverages forms) and kimchi to be among the top perceived top relievers of constipation. Interest in fermented foods is growing due to the characteristics of consuming foods with live microbes that could positively influence the gastrointestinal environment [27, 28, 29]. In the interest of the present study, a recent systematic review comprising RCTs exploring the potential efficacy of fermented foods in improving gastrointestinal function highlighted benefits to outcomes of relevance to constipation, including stool consistency, frequency, transit time, as well as gastrointestinal symptoms such as bloating, borborygmic and flatulence [30].

White bread, refined sugary breakfast cereals, red processed meat and cake were the most frequently reported contributors to constipation. In a previous survey of 122 people with chronic constipation, chocolate, bananas and black tea were the most common contributors; however, this was based on a restricted list of only seven food and drink items, limiting the range of foods assessed and potentially overlooking other relevant contributors [31]. In agreement with our findings, an observational study in 3835 Japanese women showed that higher intake of bread and confectionaries was linked to a greater odds of constipation [32]. There is a need for more research to better understand the full range of dietary contributors to constipation and inform tailored dietary recommendations in clinical care.

Gut symptoms were the outcome most preferred to be assessed in future clinical trials, followed by stool output. Interestingly, gut symptoms, such as straining, bloating, and abdominal discomfort, were also frequently reported by people with constipation as being important for a diagnosis of constipation, highlighting patients' emphasis on symptom relief beyond just stool frequency and consistency [33]. The US Food and Drug Administration recommends assessing complete spontaneous bowel movements as a primary outcome for constipation‐predominant irritable bowel syndrome [34]. This suggests that what clinicians and researchers may believe to be important outcomes to improve, for patients, a wider battery of constipation symptoms is also desired to be rectified. Indeed, out of 23 RCTs included in our previous systematic review investigating foods, drinks and diets in constipation, all 23 reported stool frequency in their outcome measures, 20 reported stool consistency, while only 10 reported straining, six reported abdominal pain and five reported incomplete evacuation. Such symptoms can significantly impact quality of life, as these symptoms are frequently experienced and can hinder daily life [2, 33, 35]. This survey suggests that symptom‐based primary outcomes, in addition to secondary stool output outcomes, may be more clinically meaningful in future studies, as they better address patients' needs and preferences.

On average, more foods were perceived to be relievers of constipation (50.7%) than to be a contributor (18.7%). Our regression results suggest that younger people with constipation report a greater number of dietary contributors compared to older people. This could relate to differences in duration of constipation experienced by the two groups or greater exposure to more diverse range of foods and therefore more contributors in younger people. It may also suggest a greater degree of dietary hypervigilance in younger generations due to increased access to dietary information and societal influences compared to older generations. Our regression models, however, did not reveal a significant difference in the number of reported dietary relievers and/or contributors based on duration of constipation.

This is the first study to comprehensively explore perceptions of the role of diet in constipation and, thus, has generated novel findings that are applicable to both clinical practice and future research. A comprehensive list of 143 foods and drinks was used to assess perception of the effects on constipation symptoms and, as such, we were able to gather a detailed picture across a wide range of dietary components for perceived relieving and contributory effects towards constipation. Several evidence‐based techniques were used in this study to increase response rates and ensure representative populations, including using a non‐monetary incentive [36]. Our study also has limitations, including a relatively small study population. In addition, the majority of our respondents were well educated, white, and female, residing in England and, therefore, may not be as representative of the wider more diverse general population with constipation. People with self‐reported constipation were surveyed, which is an important distinction, as the population requirement for seeking effective treatment is not dependent on receiving a formal diagnosis from a medical professional or from formal criteria.

Moving forward, research efforts should prioritise the evaluation of whole dietary patterns and under‐investigated individual foods to better reflect patient preferences and enhance the clinical relevance of interventions. Additionally, the incorporation of symptom‐based outcomes, such as straining and bloating, in future trials may improve the applicability of findings to real‐world experiences. These results emphasise the importance of patient‐centred research and underscore the need for tailored dietary strategies that are both evidence‐based and aligned with the experiences of those with constipation. Ultimately, such an approach may lead to more effective, acceptable, and holistic management strategies for this burdensome condition.

## 5 Conclusion

This study provides important insights into how people with constipation perceive the role of diet in managing their condition, as well as their preferences for future research directions. The findings confirm that dietary interventions are highly valued, preferring them over medication due to perceived benefits for both overall health. However, a disconnect exists between patient behaviours, the current evidence base and some clinical guidelines. The limited clinical research on whole diets and commonly perceived food contributors further highlights critical gaps that need to be addressed.

## Author Contributions

**Dominic N. Farsi:** methodology, project administration, data curation, formal analysis, visualisation, writing – original draft. **Kevin Whelan:** conceptualisation, methodology, supervision, writing – original draft, writing – review and editing. **Eirini Dimidi:** conceptualisation, formal analysis, methodology, project administration, supervision, writing – original draft, writing – review and editing.

## Funding

The authors received no specific funding for this work.

## Ethics Statement

Ethical approval was received from the King's College London Research Ethics Committee (LRS/DP‐23/24‐42020). All participants provided informed consent before participation.

## Conflicts of Interest

D.F. has no conflicts of interest to declare. K.W. has received research grants related to diet and gut health and disease from the Almond Board of California, Danone, and International Nut and Dried Fruit Council and has received speaker fees from Danone and Yakult. K.W. is the holder of a joint patent to use volatile organic compounds as biomarkers in irritable bowel syndrome (PCT/GB2020/051604). K.W. receives royalties from Wiley Publishing in relation to an academic textbook on nutrition and dietetics. E.D. has received an education grant from Alpro, research funding from the Biotechnology and Biological Sciences Research Council, British Dietetic Association, Almond Board of California, the International Nut and Dried Fruit Council and Nestec Ltd and has served as a consultant for Puratos and Danone.

## Supporting information

Supporting Figure 1. Flow diagram representing the number of questionnaires started and completed. GI, gastrointestinal; UK, United Kingdom.

Research_Priorities_Constipation_Supplementary_Figure_2_JHND.

Research_Priorities_Constipation_Supplementary_table_1_JHND.

Research_Priorities_Constipation_Supplementary_table_2_JHND.

Research_Priorities_Constipation_Supplementary_table_3_JHND_REVISED.
