# Supplementary material for: Perceptions of the Role of Diet Among People With Constipation: Dietary Contributors and Relievers to Symptoms and Research Priorities
Source: J Hum Nutr Diet. 2026 Jan 21;39(1):e70201. doi: 10.1111/jhn.70201 (PMC12820912; doi:10.1111/jhn.70201)

Supplementary Figure 1. Flow diagram representing the number of questionnaires started and completed. GI, gastrointestinal; UK, United Kingdom

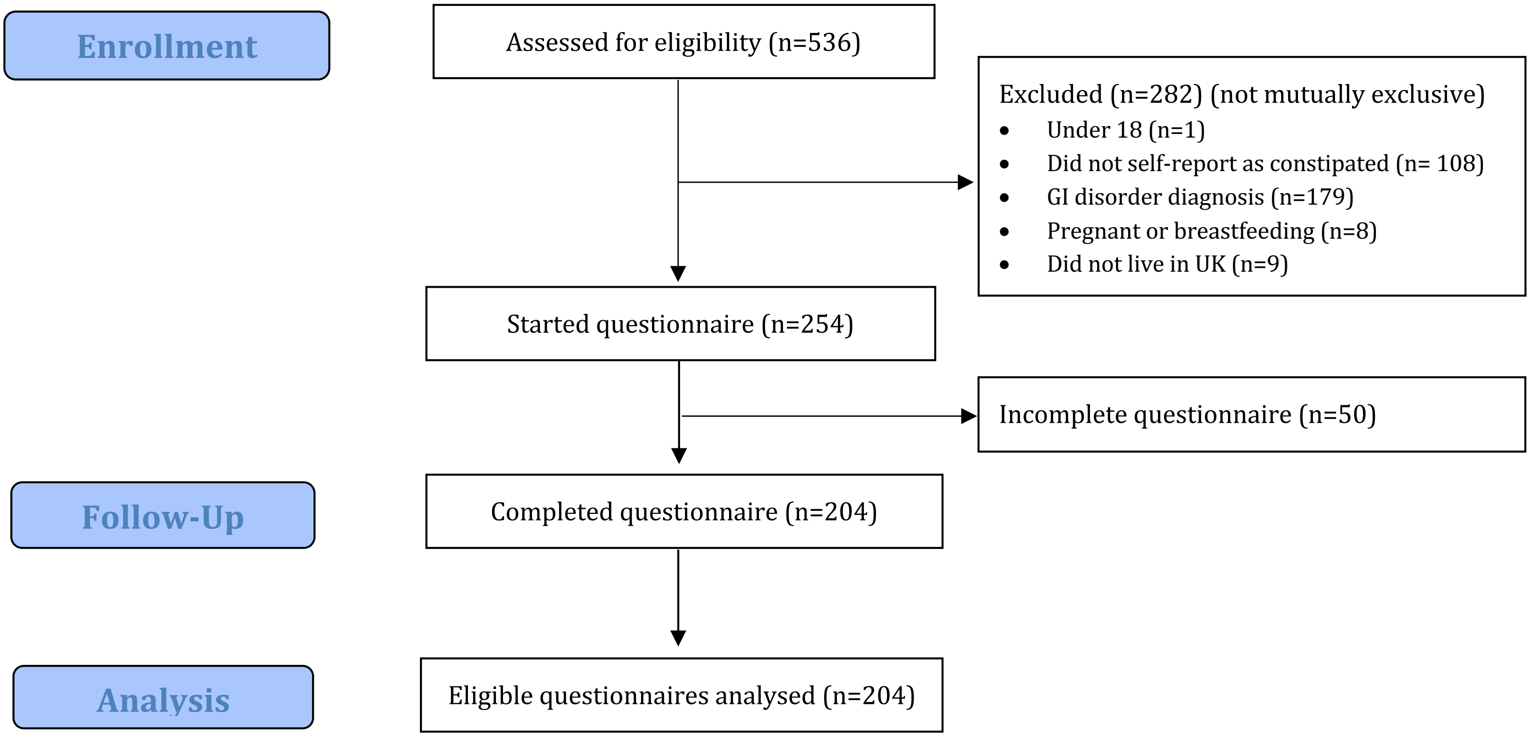

Supplement: Supplementary file 1 — Supporting Figure 1. Flow diagram representing the number of questionnaires started and completed. GI, gastrointestinal; UK, United Kingdom. [file JHN-39-0-s001.pdf]
