# Supplementary figures and images for: Perceptions of the Role of Diet Among People With Constipation: Dietary Contributors and Relievers to Symptoms and Research Priorities
Source: J Hum Nutr Diet. 2026 Jan 21;39(1):e70201. doi: 10.1111/jhn.70201 (PMC12820912; doi:10.1111/jhn.70201)

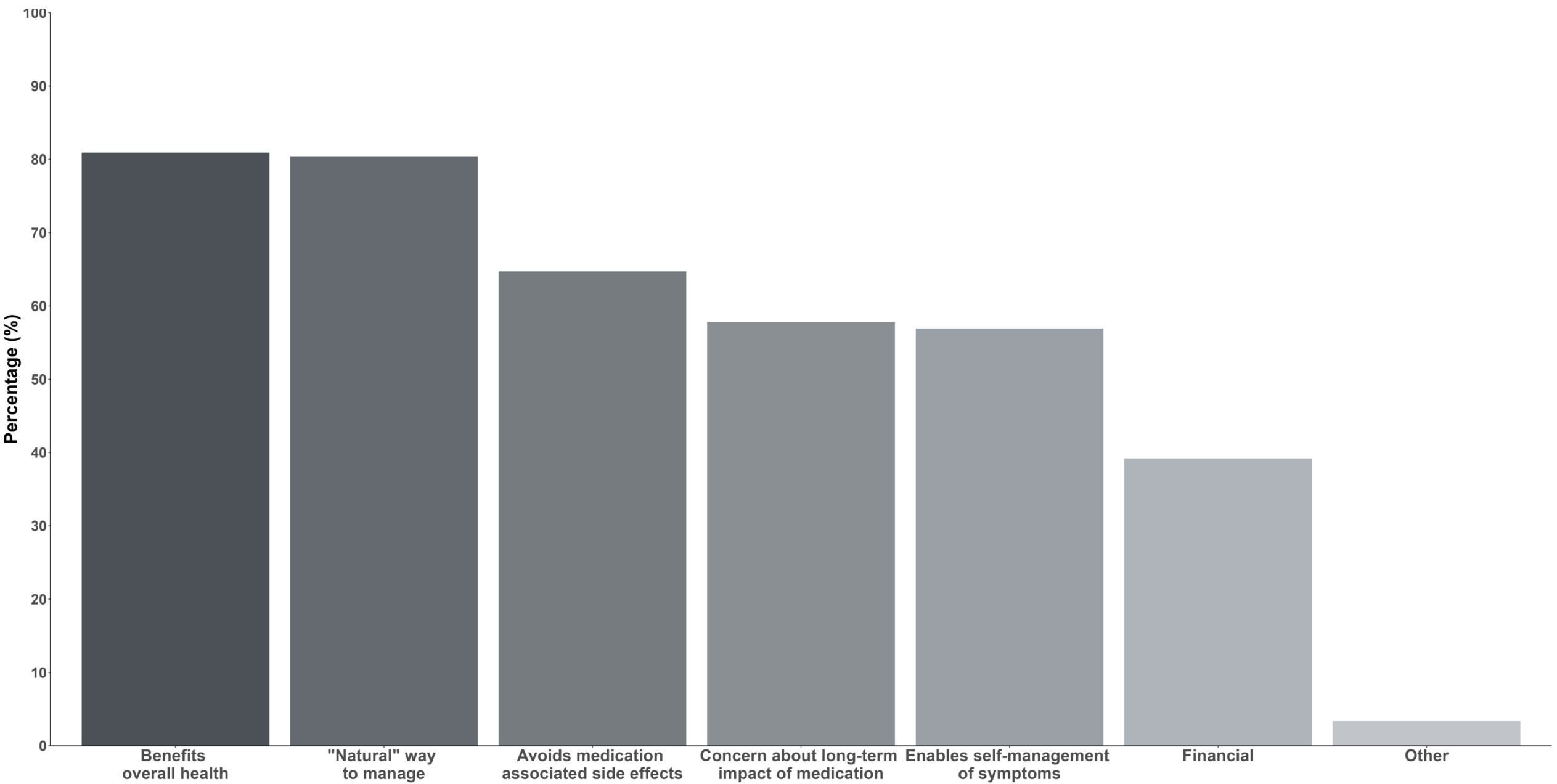

Supplement: Supplementary file 2 — Research_Priorities_Constipation_Supplementary_Figure_2_JHND. [file JHN-39-0-s003.pdf]
